# Rare Pseudopapillary Neoplasm of the Pancreas: A 10-Year Experience

**DOI:** 10.1155/2021/7377991

**Published:** 2021-09-17

**Authors:** Suvendu Sekhar Jena, Samrat Ray, Sri Aurobindo Prasad Das, Naimish N Mehta, Amitabh Yadav, Samiran Nundy

**Affiliations:** Department of Surgical Gastroenterology and Liver Transplantation, Sir Ganga Ram Hospital, Old Rajinder Nagar, New Delhi 110060, India

## Abstract

**Introduction:**

The solid pseudopapillary epithelial neoplasm (SPN) is a rare form of pancreatic neoplasm with an incidence of 2-3% of all pancreatic tumours. The recent increase in incidence is attributed to the increasing use of imaging techniques for nonspecific abdominal complaints. We report our institutional experience in the management of this tumour over the last decade.

**Method:**

We retrospectively analyzed from a prospectively maintained database of patients from January 2011 to December 2020 who were operated upon for SPN. All the patients were followed till date.

**Results:**

Of 479 patients operated on for various types of pancreatic tumours during this period, 15 (3.1%) had SPN. The mean age of presentation was 28 years with a female preponderance (12/15, 80%). The most common location was the body and tail of the pancreas (66%), and the mean size was 6.4 cm (2–15 cm). The tumour extent was defined as ‘borderline resectable' in 20% of cases. Distal pancreatectomy was done in 11 patients with spleen preservation in 3. *R*0, *R*1, and *R*2 resection were done in 12, 2, and 1 patient(s), respectively. The operative mortality was 6.7%. All the patients are doing well on follow-up.

**Conclusion:**

SPN is a low-grade malignant tumour with a strong female predilection. Clinical manifestations have no specificity, imaging examination only contributes tumour location, and the final diagnosis rests on pathology. Surgery is the main modality of treatment and carries a good prognosis.

## 1. Introduction 

Solid pseudopapillary neoplasms (SPNs) of the pancreas are defined by the new 5th edition of the World Health Organization (WHO) Classification of Digestive System Tumors as low-grade malignant tumours composed of poorly cohesive epithelial cells which form solid and pseudopapillary structures and lack a specific line of pancreatic epithelial differentiation [1]. SPN incidence is around 2-3% of all pancreatic neoplasms. It has a female preponderance and also malignant potential [2]. A total of 8334 cases have been reported in the English literature till 2018 with an increasing incidence in recent times. This is attributed to the extended use of imaging techniques for nonspecific abdominal complaints and their better availability since most of the tumours are indolent and patients have nonspecific complaints [3]. Cubilla and Fitzgerald reported an incidence of SPN of 0.17% in 1979, Morohoshi et al. reported an incidence of 2.7% in 1987, and Koshmal et al. reported an incidence of 6.1% of all pancreatic tumours in 2004 [4–6]. Lichtenstein was the first to report this entity, but Frantz in 1959 described its pathology and Hamoudi et al. described its electron microscopy features in 1970 [7–9]. Before being defined as a tumour of uncertain differentiation or solid pseudopapillary neoplasm in 1996 by the World Health Organization, it was also known as a solid cystic tumour, papillary epithelial neoplasm, or papillary epithelial tumour [10]. It is a rare tumour and has an estimated incidence of 2-3% of all pancreatic tumours and 6–12% of all pancreatic cystic neoplasms [3]. It usually affects women below the age of 40 years (2–85 years), with a male to female ratio of 7–11 : 1. In the paediatric age group, the incidence is 8–12.5%. The usual location is the body and tail of the pancreas (55–60%) followed by the head and neck of the gland (35–40%). It is usually associated with a favourable prognosis with a long-term disease-free survival of 95% [11]. SPNs are slow-growing tumours and have a low malignant potential, and the usual symptoms are due to local manifestations of the tumour. It may occasionally present with tumour rupture or metastasis. The origin of the disease is unknown with various authors claiming different points of origin. Surgery is the mainstay of treatmentwhich depends upon the location of the tumour and ranges from distal pancreatectomy and Whipple's pancreaticoduodenectomy, to debulking. Radiotherapy and chemotherapy have very little or no role in its management [12]. We had previously published a case report of a young girl presented with ruptured SPN in 2016 and highlighted various aspects of management [13]. The present study was done to report our institutional experience in the management of this rare tumour over the last decade.

## 2. Patients and Methods

We retrospectively analyzed, from a prospectively maintained database, all patients with a diagnosis of solid pseudopapillary epithelial neoplasm operated at the Department of Surgical Gastroenterology and Liver Transplantation at Sir Ganga Ram Hospital, New Delhi, over a period of 10 years from January 2011 to December 2020. Their demographic data, associated comorbidities, presenting complaints, preoperative blood parameters, and preoperative tumour markers like carcinoembryonic antigen (CEA) and carbohydrate antigen 19-9 (CA 19-9) were analyzed. The imaging results, i.e., the ultrasound and contrast-enhanced computed tomography (CECT) of the abdomen, were also analyzed. The type of surgery performed and its complications along with the histopathology reports were also recorded, and the complications were graded according to the Clavien–Dindo classification (CDC) and comprehensive complication index (CCI) [14, 15]. The postoperative pancreatic fistula (POPF) was defined as per the definition of an international study group of pancreatic fistula. The patients were followed up till December 2020. The pancreatic exocrine functions were assessed from the symptoms and need for pancreatic enzyme supplementation, while the endocrine functions were assessed by the need for insulin or any increase in the requirement of insulin if already on insulin. The data were compared with those published from western countries as well as from India. Statistical analysis was performed using SPSS version 22.0 software. Continuous variables were reported as mean ± standard deviation (±SD). Informed consent was taken from the patients for the use of their data.

## 3. Results

Of 479 patients operated for various types of pancreatic tumours during this period, 15 (3.1%) were operated for SPN. They had a mean age of 28.13 (±11.2) years (11–47). Of 15 patients, 12 were female and 3 were male. Their mean body mass index (BMI) was 22.37 (±3.85) kg/meter^2^. Only 1 patient had hypertension as an associated comorbid illness with a mean Charlson comorbidity index of 8.7.

The majority of the patients presented with a mild, dull aching type of pain (66.7%), while one patient was incidentally diagnosed while being evaluated for unexplained anaemia when she was found to have a SPN along with a uterine fibroid. One patient presented with severe abdominal pain and on evaluation was found to have a ruptured SPN. Three patients presented with a lump in the abdomen. The median size of the tumour was 6 cm (2–15 cm). The location was the body and tail of the pancreas in 5, tail in 5, and body in 3, 1 each in head and uncinate process (Table 1).

**Figure 1:**
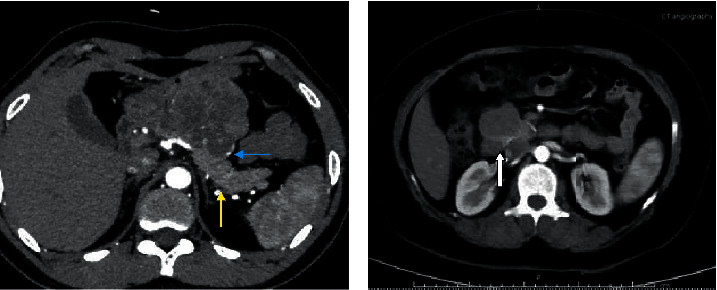
(a) CECT abdomen showing an exophytic growth arising from the body and neck of the pancreas (yellow arrow) displacing the stomach with a peripheral curvilinear calcification (blue arrow). (b) Exophytic growth arising from the head of the pancreas (white arrow).

**Figure 2:**
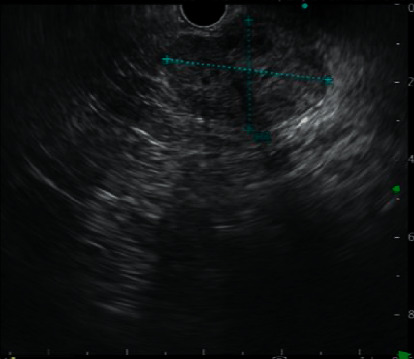
A 3 × 4 cm lesion with a small cystic component arising from the uncinate process of the pancreas.

Preoperative tumour markers like the CA 19-9 and CEA, found to rule out primary pancreatic adenocarcinoma or metastatic disease, were normal in all cases. The imaging modalities used were ultrasound (USG) abdomen and CECT abdomen. USG abdomen was done in 12 patients, among whom the tumour was hypoechoic in 5, heteroechoic in 5, and isoechoic in 2 patients. CECT abdomen revealed a borderline resectable lesion in 3 patients and resectable lesion in 12 patients. The tumour was solid only in 8 patients, cystic only in 1 patient, and mixed solid and cystic in 6 patients. The cystic areas probably indicated haemorrhage into the tumour. In 14 cases, the tumours were encapsulated. Calcification was seen in 12 patients (Figure 1). Endoscopic ultrasound (EUS) was done as a part of the diagnostic procedure in 6 patients (Figure 2). 2 patients had a preoperative diagnosis other than SPN on EUS, like islet cell tumour in one patient and pancreatic cystadenoma in the other. A preoperative biopsy was done in 8 patients, among whom 6 had fine-needle aspiration cytology via EUS and 2 underwent exploration and biopsy somewhere else for a lump in the abdomen before coming here (Table 2).

**Figure 3:**
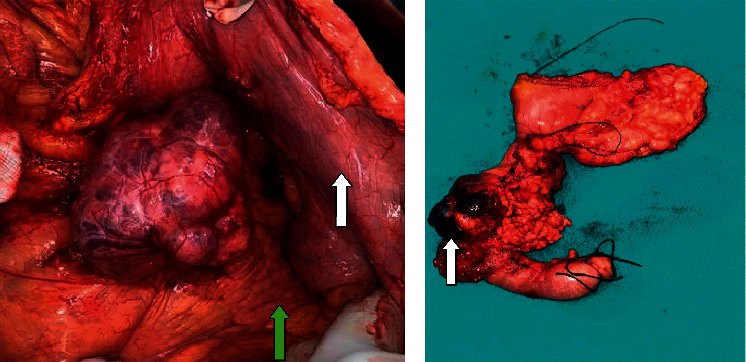
(a) Exophytic growth arising from the body and neck of the pancreas with reflected stomach anteriorly (white arrow) and normal pancreas (green arrow). (b) Whipple's pancreaticodudoenectomy specimen; exophytic growth arising from the head of the pancreas with cystic areas (white arrow).

All 15 patients underwent curative resection. The patient who presented with a tumour rupture underwent emergency laparotomy due to haemodynamic instability and altered sensorium. Upon exploration, around 1400 ml of blood was found in the peritoneal cavity. The mass was found to be adherent to the splenic flexure of the colon, spleen, and transverse mesocolon with complete obliteration of the lesser sac. Because of her unstable condition, peritoneal lavage and biopsy for the tumour were performed. She then received four cycles of chemotherapy using vincristine (1.5 mg/m^2^), actinomycin D (0.045 mg/kg), and cyclophosphamide (1200 mg/m^2^) to reduce the size of the tumour. The tumour response was assessed with the USG abdomen. She subsequently underwent distal pancreaticosplenectomy. This procedure was performed in a total of 11 patients with spleen-preserving distal pancreatectomy in 3 patients. Whipple's pancreaticodudoenectomy was performed in 2 patients. One patient had a central pancreatectomy with Roux-en-Y reconstruction (Figure 3). None of the patients underwent vascular resection or reconstruction. Of 11 patients who underwent distal pancreatectomy with or without splenectomy, 6 patients underwent laparoscopic resection while 5 underwent open resection. Multivisceral resection was not required in any of the patients. The average blood loss was 228 ml. A total of 4 patients received blood transfusion. The average duration of surgery was 268.3 ± 145.8 min (Table 3).

**Figure 4:**
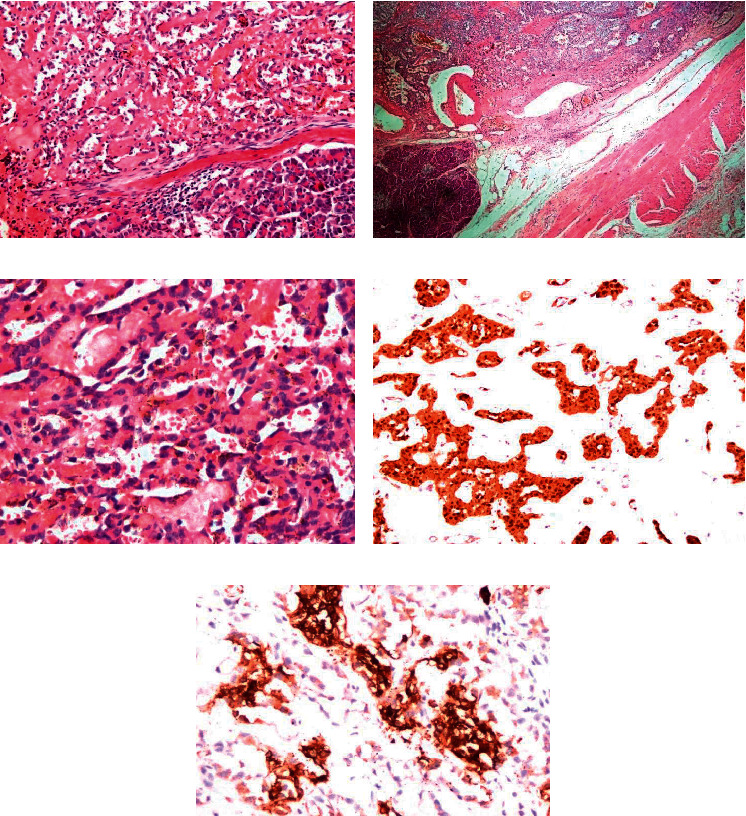
(a) Tumour cells arranged as solid nests and pseudopapillae with intervening hyalinised vascular channels (normal pancreas) (20x). (b) Tumour appears fairly delineated, however, in places it is intermixed with pancreatic acini at the periphery. The tumour is abutting the superficial fibres of the muscularis propria of the duodenum. (c) The tumour cells are polyhedral with mildly anisomorphic nuclei having longitudinal grooves, inconspicuous nucleoli, and moderate to scant amount of eosinophilic cytoplasm (40x). (d) Beta-catenin +. (e) Synaptophysin +.

R0 resection was performed in 12 patients, while *R*1 resection was performed in 2 patients. The patient, who presented with tumour rupture, initially underwent damage control surgery (*R*2) followed by tumour downsizing with chemotherapy and complete resection. None of the patients had lymphovascular invasion, while 1 of them showed perineural invasion. On immunohistochemical (IHC) staining, cells were stained positive for vimentin in 13 patients and synaptophysin and beta-catenin in 11. Chromogranin was negative in all 15 patients (Table 4) (Figure 4). The Ki-67 proliferation index was less than 1% in all cases. The average number of lymph nodes retrieved were 10 ( 2–24), and none of the nodes showed metastatic deposits.

There was 1 patient with operative mortality. The patient underwent exploratory laparotomy, cholecystectomy, gastrojejunostomy, and biopsy of intra-abdominal mass elsewhere following which there was a bile leak followed by endoscopic stenting of the common bile duct. He then received 3 cycles of chemotherapy and underwent distal pancreaticosplenectomy in our hospital. He had a POPF that was managed conservatively, and he was discharged with a drain in situ. Gradually, the drain output reduced and it was removed. He was readmitted with hematemesis and hemorrhagic shock on postoperative day (POD) 45. On evaluation, there was a pseudoaneurysm of the gastroduodenal artery which was managed by angioembolization. He subsequently developed septicaemia and died on POD 88.

All the patients were followed up. At a median follow-up of 59 months (2–109), there was no recurrence. Except for the patient who underwent damage control surgery, no patient received adjuvant chemotherapy. There was no endocrine or exocrine insufficiency in any of the patients.

## 4. Discussion

SPN is a rare pancreatic tumour, accounting for 1-2% of all pancreatic neoplasms [16]. Unlike pancreatic adenocarcinoma, these tumours are indolent and benign with a risk of malignant transformation in around 10–15% [17]. Their cell of origin is unknown. One theory suggests it originates from multipotent primordial germ cells lacking a definite differentiation of the exocrine or endocrine cell [18]. Various authors have also described various sources of origin like the acinar cell and endocrine cell. Another theory suggests its origin from the incorporation of primitive ovarian cells within the pancreatic parenchyma during the seventh week of embryogenesis, but this does not explain its occurrence in males [19–22].

It is frequently seen in young females in their second to fourth decade of life with a female preponderance of 80% and mean age of 28.13 ± 11.2 years. Law et al. in their systematic review of 2744 patients revealed 87.8% incidence in females with an average age of 28.5 ± 13.7 years [23].

Obesity had no relation with SPN in our study which was also confirmed by Song et al. with an average BMI of 23.7 ± 2.4 kg/m^2^ [8]. The average BMI in our study was 22.37 ± 3.85 kg/m^2^ (14.3–27.8). There are some reports of an association between hepatitis B virus and SPN due to overexpression of beta-catenin in tumour cells, but none of our patients had hepatitis B [24].

The symptoms are mostly nonspecific, and abdominal pain caused by the mass is the most common manifestation with nausea and vomiting being other symptoms [3]. The subtle nature of their presentation leads to a delay in diagnosis, resulting in large tumours found at that time. However, the size of the lesion does not affect resectability [25]. SPNs are usually bulky tumours (median tumour size 6 cm in our study). Despite their large size, they usually do not invade the surrounding structures but only displace them, so symptoms like obstructive jaundice and pancreatitis are rare. Abdominal pain was the most common mode of presentation seen in 66.7% of our cases followed by a lump in 20%. Incidental detection was seen in one. Usually, it is restricted to the pancreas in approximately 85% of cases, while 10–15% of cases present with metastases [23]. The most common sites of metastasis are the liver, regional lymph nodes, omentum, and peritoneum [26]. None of our patients had metastasis during their presentation.

Although rare, rupture of the tumour can also be a mode of presentation that can either be spontaneous or following blunt trauma to the abdomen. The reported incidence of rupture in the literature is 2.7% with blunt trauma to the abdomen being the most common cause [27]. The incidence of spontaneous rupture is 1% of all SPN and thought to be due to sudden massive haemorrhage causing a rise in pressure inside. One 11-year-old young girl presented to us with a sudden onset of abdominal pain with haemodynamic instability and underwent debulking because of bleeding and infiltration of the surrounding structures.

The most common location of the tumour was the body and tail of the pancreas in 80% of cases. Law et al. in their meta-analysis showed 59.3% located in the body and tail of the pancreas [23], and one of the largest meta-analyses conducted by Yao et al. in 2450 Chinese patients also showed the most common location to be the body and tail of the pancreas. In contrast, Panieri et al. showed an equal spatial distribution of the tumour [28, 29]. The size of the tumour or its location did not predict malignancy as reported by Yu et al. [30]. Ectopic locations have also been described in the retro peritoneum, mesentery, and left adrenal gland [28]. None of our patients had an ectopic location.

No additional information in our series was provided by the haematological investigations, while hyperamylasaemia, elevated hepatic enzymes, and leukocytosis were occasionally identified [24]. In tumour markers like carbohydrate antigen 19-9 and carcinoembryonic antigen did not contribute to the diagnosis. In preoperative assessment, radiological studies are the most important. Abdominal roentgenograms can demonstrate displacement of the stomach, colon, or spleen by an extrinsic mass [31]. Calcification is seldom encountered within the large mass. However, calcifications are peripheral and curvilinear when present, as compared to the pattern of sunburst defined in microcystic adenomas [32]. Ultrasonography, CT scan, and MRI typically demonstrate the same characteristics with an encapsulated tumour composed of solid and cystic elements, often with capsule rim-like calcifications as well as intraparenchymal calcifications. The lesion has well-defined margins often without pancreatic duct dilation. Ultrasonography demonstrates hypoechoic, isoechoic, or heteroechoic components, while CT shows solid or cystic components with calcification. MRI and positron emission tomography can also be used. MRI is better than CT in various aspects like demonstration of a capsule and solid or cystic degeneration [33]. None of the radiological findings are specific for SPN, and similar findings can also be seen in other cystic neoplasms and pancreatoblastoma. Endoscopic ultrasound and fine-needle aspiration were used in 40% of cases. Preoperative tissue diagnosis is not always necessary but can be used in cases of uncertain diagnosis [12].

Distal pancreatectomy with or without splenectomy was performed in 11 (73.3%) cases, and Whipple's pancreaticoduodenectomy was performed in 2 (13.3%) patients. Although no patients in our study required vascular resection, various studies have shown excellent long-term survival after vascular or multivisceral resection [34, 35]. Extensive lymphadenectomy is not done always due to the low incidence of lymph node metastases [12]. No patient showed lymphovascular invasion in our study, and all the resected nodes were negative for tumour deposits. Metastatectomy also improves long-term outcomes [12]. Debulking of a locally advanced tumour also has a role as shown by a previous case report from our institution [13]. Enucleation is also another option for moving small tumours away from the duct but carries a risk of fistulae and tumour dissemination. Complete *R*0 resection was performed in 80% of patients, while 13.3% had an *R*1 resection. It is difficult to differentiate between the malignant and benign varieties as the criteria for malignancy is not clear cut. As per the WHO classification, the clear-cut criteria for malignant varieties are lymphovascular or perineural invasion and liver or lymph node metastases which are known as pseudopapillary carcinoma. A size of more than 5 cm, capsular invasion, high Ki-67 proliferation index, and growth into peripancreatic tissue are also linked with malignancy by a few authors [36]. Nishihara et al. proposed venous invasion, high nuclear grade, and prominent necrobiotic nests as indicators of malignancy [37]. Only one patient had a perineural invasion in our study and is surviving 5 years after surgery without any recurrence. All our patients had a low Ki-67 proliferation index of <1%.

The most common complication following surgery was postoperative pancreatic fistula, probably due to the soft nature of the pancreas, which was seen in 8 (53.3%) patients of which grade A fistula, that is a biochemical leak, was seen in 6 patients while grades B and C were seen in one patient each. There was one patient with mortality having grade C fistula, following distal pancreaticosplenectomy due to sepsis and haemorrhage from a gastroduodenal artery pseudoaneurysm on POD 88.

On histopathology, SPN is a cellular neoplasm with cells arranged in several layers around a fibrovascular stalk giving a pseudopapillary appearance. The presence of a pseudopapillary structure, hyaline globules, cholesterol clefts, foamy macrophages, and nuclear grooving in the absence of salt-pepper chromatin on histopathology are characteristics of SPN. Immunohistochemical markers are also peculiar to SPN and can be used to differentiate it from various conditions like cystic neoplasms of the pancreas, pancreatoblastoma, acinic cell tumour, and neuroendocrine tumours [36]. Beta-catenin and Wnt signaling pathway have been found to play an important role in the tumorigenesis and are consistently positive in 90% of cases of SPN, which is consistent with our study where beta-catenin was found to be positive in 86.6%. The other consistently positive markers were vimentin, neuron-specific enolase, and *α*1-antitrypsin. The neuroendocrine markers, except chromogranin A which is consistently negative in SPN, like synaptophysin, neuron-specific enolase, and CD 56 also showed variable expression [37, 38]. The positive expression of the progesterone receptor was also seen in 53.35% of patients suggesting an origin from ovarian cells.

The literature regarding the use of adjuvant chemotherapy is limited with various authors trying gemcitabine, oxaliplatin, irinotecan, and etoposide in various combinations postoperatively and reporting disease stability. Various authors also used chemotherapy to downstage the tumour and in turn facilitated surgery and prolonged survival. Radiation can be used in case of an unresectable tumour to provide a better quality of life and even a survival benefit. Selective internal chemotherapy has also been described. Tamoxifen is a viable option for metastatic disease in patients who are poor candidates for chemotherapy [39]. In our study, one patient who had presented with tumour rupture underwent debulking initially due to her haemodynamic instability, received chemotherapy, and underwent definitive resection at a later stage after downstaging of her tumour.

The overall five-year survival rate after surgery is around 95–97% [40]. According to Yu et al., the estimated 1-, 3-, and 5-year survival rates were 99.4%, 97.5%, and 96.9%, respectively [41]. The tumour recurrence rate was <10% [42]. In our study, at a median follow-up of 59 months (2–109), there was no recurrence of the tumour and none of the patients has signs of exocrine or endocrine insufficiency.

## 5. Conclusion

To conclude, although rare, SPN are treatable tumours, the origin of which is still an enigma. Mostly seen in young females, they have a nonspecific presentation. They can be readily identified by radiological imaging, while typical histological and immunochemical markers separate these from their close counterparts. Because of the risk of malignant transformation and for good survival, resection is the best option. The size of the tumour does not predict the outcome. Locally advanced tumours can be given chemotherapy to downstage them for a definitive resection. Aggressive lymphadenectomy is not required.

## Figures and Tables

**Table 1:** Demographic variables.

Variable	*n* = 15
Age in years (mean + SD)	28.13 ± 11.20

*Sex*	
Male	3
Female	12

BMI (kg/m^2^)	22.37 ± 3.85

*Presentation*	
Symptomatic	14
Incidentally	1

*Presenting symptoms*	
Pain	113
Lump abdomen	1

*Incidental*	
Tumour size in cm (median)	6.4 (2–15)

*Location of tumour*	
Body	3
Tail	5
Body and tail	5
Head	1
Uncinate process	1

SD: standard deviation; BMI: body mass index; *n*: number of patients.

**Table 2:** Diagnostic modalities and findings.

Diagnostic modalities	Incidence (*n*)
*Ultrasound (n* *=* *12)*	
Hypoechoic	5
Heteroechoic	5
Isoechoic	2

*CECT abdomen (n* *=* *15)*	
Borderline resectable	3
Resectable	12
Encapsulated	14
Calcification	12

*EUS*	
Performed	6

*Preoperative suspicion*	
SPN	13
Others	2

*Preoperative biopsy*	
Performed	8
EUS guided	6
Exploratory laparotomy	2
Not performed	7

**Table 3:** Operative parameters.

Variables	*n* = 15
*Surgery performed*	
Distal pancreatectomy	11
Splenectomy	8
Spleen preservation	3
Whipple PD	2
Central pancreatectomy	1
Debulking	1
Laparoscopic resection	6
Open resection	9
Blood loss (in ml)	228
Duration of surgery (min)	268.3 ± 145.8
Blood transfusion	4
Hospital stay (days)	6 (3–16)

*Complication*	
Clavien–Dindo grading	10
I	5
II	5
III	0
IV	0
Comprehensive complication index	8.7 (0–36.2)
Postoperative pancreatic fistula	8
Grade A	6
Grade B	1
Grade C	1
Mortality	1

**Table 4:** Immunohistochemical markers.

IHC marker	Positive (n)	Negative (n)
Vimentin	13	2
Beta-catenin	11	4
Synaptophysin	11	4
CD 10	9	6
PR	8	7
Cytokeratin	6	9
NSE	5	10
CEA	4	11
Chromogranin	0	15

PR: progesterone receptor; NSE: neuron-specific enolase; CEA: carcinoembryonic antigen.

## Data Availability

Data are available with the corresponding author and can be reproduced upon request.
